# P-ODN: Prototype-based Open Deep Network for Open Set Recognition

**DOI:** 10.1038/s41598-020-63649-6

**Published:** 2020-04-28

**Authors:** Yu Shu, Yemin Shi, Yaowei Wang, Tiejun Huang, Yonghong Tian

**Affiliations:** 1 School of Academy for Advanced Interdisciplinary Studies, Peking University, Beijing, China; 2 Department of Computer Science and Technology, Peking University, Beijing, China; 3 National Engineering Laboratory for Video Technology, School of EECS, Peking University, Beijing, China; 4 Peng Cheng Laboratory, Shenzhen, China

**Keywords:** Computational science, Computer science

## Abstract

Most of the existing recognition algorithms are proposed for closed set scenarios, where all categories are known beforehand. However, in practice, recognition is essentially an *open set* problem. There are categories we know called “knowns”, and there are more we do not know called “unknowns”. Enumerating all categories beforehand is never possible, consequently, it is infeasible to prepare sufficient training samples for those unknowns. Applying closed set recognition methods will naturally lead to unseen-category errors. To address this problem, we propose the prototype-based Open Deep Network (P-ODN) for open set recognition tasks. Specifically, we introduce prototype learning into open set recognition. Prototypes and prototype radiuses are trained jointly to guide a CNN network to derive more discriminative features. Then P-ODN detects the unknowns by applying a multi-class triplet thresholding method based on the distance metric between features and prototypes. Manual labeling the unknowns which are detected in the previous process as new categories. Predictors for new categories are added to the classification layer to “open” the deep neural networks to incorporate new categories dynamically. The weights of new predictors are initialized exquisitely by applying a distances based algorithm to transfer the learned knowledge. Consequently, this initialization method speeds up the fine-tuning process and reduce the samples needed to train new predictors. Extensive experiments show that P-ODN can effectively detect unknowns and needs only few samples with human intervention to recognize a new category. In the real world scenarios, our method achieves state-of-the-art performance on the UCF11, UCF50, UCF101 and HMDB51 datasets.

## Introduction

Deep neural networks have demonstrated significant performance on many visual recognition tasks^1–3^. Almost all of them are proposed for closed set scenarios, where all categories are known beforehand. However, in practice, some categories can be known beforehand, but more categories can not be known until we have seen them. We call the categories we know as priori the “knowns” and those we do not know beforehand the “unknowns”. Enumerating all categories is never possible for the incomplete knowledge of categories. And preparing sufficient training samples for all categories beforehand is time and resource consuming, which is also infeasible for unknowns. Consequently, applying closed set recognition methods in real scenarios naturally leads to unseen-category errors. Therefore, recognition in the real world is essentially an open set problem, and an open set method is more desirable for recognition tasks.

**Figure 1:**
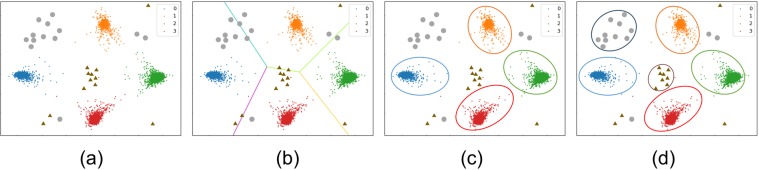
Open Set Recognition. The data set contains both knowns and unknowns, figure (**a**) shows the feature distribution that might be obtained after representation learning, where gray circles and brown triangles indicate the unknowns. Figure (**b**) shows the learned partition of the “whole” feature space, the unknowns are still predicted to some specific regions under the partition. The solution to the open set recognition should be able to accept and classify knowns into correct known categories and also reject unknowns, as shown in figure (**c**). Simultaneously, it is natural to further extend to perform recognition of unknowns as shown in figure (**d**).

To give an intuition of open set recognition problem, Fig. 1 shows the difference between closed set recognition and open set recognition from the perspective of representation learning. Assuming that a data set contains four known categories, and with the open set condition, the data set might also contain unknowns. Then after representation learning, a feature distribution as shown in Fig. 1(a) might be obtained, where gray circles and brown triangles indicate the unknowns. As the closed set recognition learns a partition of the “whole” feature space, as shown in Fig. 1(b), the unknowns are still predicted to some specific regions under the partition. The solution to the open set recognition should be able to accept and classify knowns into correct known categories and also reject unknowns, as shown in Fig. 1(c). Simultaneously, it is natural to further extend to perform recognition of unknowns as shown in Fig. 1(d). Our work is conducted on the extended setting of open set recognition like Fig. 1(d), which performs unknowns detecting at the first stage and then unknowns classifying at the second stage, which is also called Open world recognition in^4^.

Technically speaking, many methods on incremental learning can be used to handle new instances of known categories^5–9^. However, most of these approaches do not consider unknowns or dynamically adding new categories to the system. In^10^, a discriminative metric is learned for Nearest Class Mean (NCM) classification on the knowns, and new categories are added according to the mean features. This approach, however, assumes that the number of known categories is relatively large. An alternative multi-class incremental approach based on the least-squares SVM has been proposed by Kuzborskij *et al*.^11^ where for each category a decision hyperplane is learned. However, in this way, every time a category is added, the whole set of the hyperplanes will be updated again, which is too expensive as the number of categories growing.

In particular, most of the researches on open set recognition focus on detecting unknowns only, recent works^12–14^ have established formulations of classifying knowns and rejecting unknowns. While it is natural to extend to classify unknown samples after detecting unknowns. And in the real world, solutions that further classify the unknowns are more challenging and have a wider range of applications. Abhijit *et al*.^4^ proposed an SVM-based recognition system that could continuously recognize new categories in an open-world model by extending the NCM-like algorithms^15^ to a Nearest Non-Outlier (NNO) algorithm. But it is not applicable in deep neural networks, and the performance is much worse than deep neural network based algorithms. Recently, in the work^16^, Yang *et al*. have tried to handle the open set recognition problem by training prototypes to represent the unknowns. But the solution works on the assumption that all unknowns have sufficient labeled samples to train discriminative prototypes, which is not realistic. And the system needs to be retrained when new categories come, which is time and computational resource consuming.

In our previous work^17^, we proposed an Open Deep Network (ODN) algorithm for open set recognition. First, we train a CNN network to classify the knowns which have sufficient samples. Then the triplet threshold of each category is calculated based on the correctly classified features of the training set. Unknowns can be detected by applying the triplet thresholds on the features derived by the CNN. Manual labeling the unknowns which are detected in the previous process and predictors of the classification layer are added dynamically to incorporate new categories. Weights of the new predictors are initialized by applying the emphasis initialization method which transfers the learned knowledge of the CNN to speed up the fine-tuning. However, the triplet thresholds are calculated on the sampled features of the training set, consequently, the unknowns detection process might be affected by the outliers of the training set. Besides, relations of categories are defined on the feature scores in emphasis initialization method, which is a simple way to estimate the similarity of categories.

Most recently, the prototype learning was introduced to improve the robustness of CNNs. Yang *et al*. proposed the CPL to improve the robustness by using prototypes and proposed the PL (prototype loss) to improve the intra-class compactness and inter-class distance of the feature representation in the work^16^. Yang *et al*. also introduced a method to handle the open set recognition problem by using prototypes in their paper. However, as mentioned before, this method assumes that samples of unknowns are sufficient to train the prototypes. And when new unknowns come, the system needs to be retrained again. Inspired by the prototype learning concept, we propose the prototype-based Open Deep Network (P-ODN) to handle the open set recognition problem.

In this paper, we propose P-ODN to improve the robustness in detecting unknowns and updating deep neural networks, consequently facilitating open set recognition. Basically, prototypes and prototype radiuses are trained jointly to derive more precise features to better represent categories. In the *prototype module*, prototypes are taught to learn the centers of knowns. And in the *prototype radius module*, values of prototypes are further restricted to a certain range by learning a radius for each category as a regularization item of prototypes. Both of the modules help to improve the intra-class compactness and inter-class distance of the feature representation. Then the correctly classified features of the training set are projected into a different feature space by calculating the distance distribution between the features and prototypes. The triplet thresholds are learned based on the correctly classified distance distribution. Instead of detecting unknowns directly on the features, based on the statistic information of training samples, detecting unknowns based on the distance of prototypes keeps knowledge of the model, which has less potential to be affected by the outliers of the training set. After manually labeling the unknowns which are detected in the previous process, new predictors are initialized based on the distance distribution of new samples and prototypes. Each weight column of the knowns is integrated to initialize the new weight according to the distance distribution. And the distance distribution contains more robust relation knowledge of the new category and knowns. Finally, fine-tuning the model with the manual labeled samples to incorporate new categories.

In order to give a convincing result of our P-ODN, in this paper, we choose to focus on the action recognition problem which is a more challenging recognition task. The effectiveness of the proposed framework is evaluated on four public datasets: UCF11, UCF50, UCF101, and HMDB51. The experimental results show that our method can effectively detect unknowns and needs only few samples with human intervention to recognize a new category. And our method achieves the state-of-art performance on all the four datasets in real -world scenarios.

## Overview

**Figure 2:**
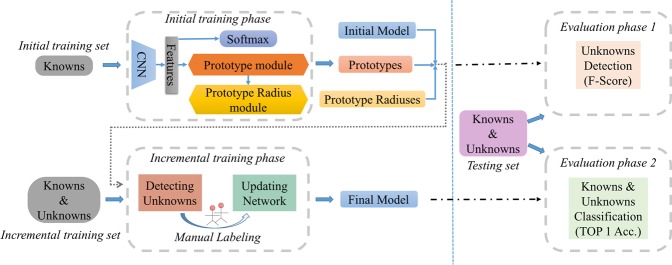
Framework of open set recognition. The left part of the blue dotted line illustrates the two training phases, the initial training phase, and the incremental training phase. The initial training phase takes the initial training set (contains the knowns only) as input, then learns and outputs an initial model, prototypes and prototype radiuses for each category. And the incremental training phase takes the incremental training set (contains both knowns and unknowns) and the outputs of the initial training phase as inputs, then detects the unknowns. Manual labeling the unknowns which are detected in the previous process. Next, the new category is dynamically incorporated in the model. Finally, fine-tuning the model with only a few samples to make the unknowns known. The right part of the dotted line illustrates two evaluation phases responding to the two training phases, the evaluation phase 1 and the evaluation phase 2. In the evaluation phase 1, the detection f-score of unknowns is measured here on the initial model trained in the initial training phase. And in the evaluation phase 2, the classification accuracy of both knowns and unknowns is measured here on the final model trained in the incremental training phase.

The framework of our open set recognition approach is shown in Fig. 2. Two training phases and two evaluation phases constitute the whole framework. The initial training set which contains only knowns is provided to the initial training phase as input. Then an initial model is trained, as well as prototypes and prototype radiuses of categories. The incremental training phase takes the incremental training set (contains both knowns and unknowns) as input and extracts the features by using the initial model. Then distances of the features and the prototypes are measured under the constraint of the prototype radiuses. Next, a triplet thresholding method proposed in our previous work^17^ is modified to apply in our framework to detect the unknowns. Manual labeling the unknowns as new categories which are dynamically incorporated in the model. Then fine-tuning the model with only a few samples to make the unknowns known. Finally, the final output model can classify both knowns and unknowns.

Two evaluation phases are set to evaluate the model performance. The evaluation phase 1 is carried out after the initial training phase. The testing set which contains both knowns and unknowns is provided to the initial model, and we measure the detection f-score of unknowns at this phase. The evaluation phase 2 is carried out after the incremental training phase. Classification TOP 1 accuracy of both knowns and unknowns is measured here as the most important performance indicator of open set recognition tasks.

## Prototype-based Open Deep Network

**Figure 3:**
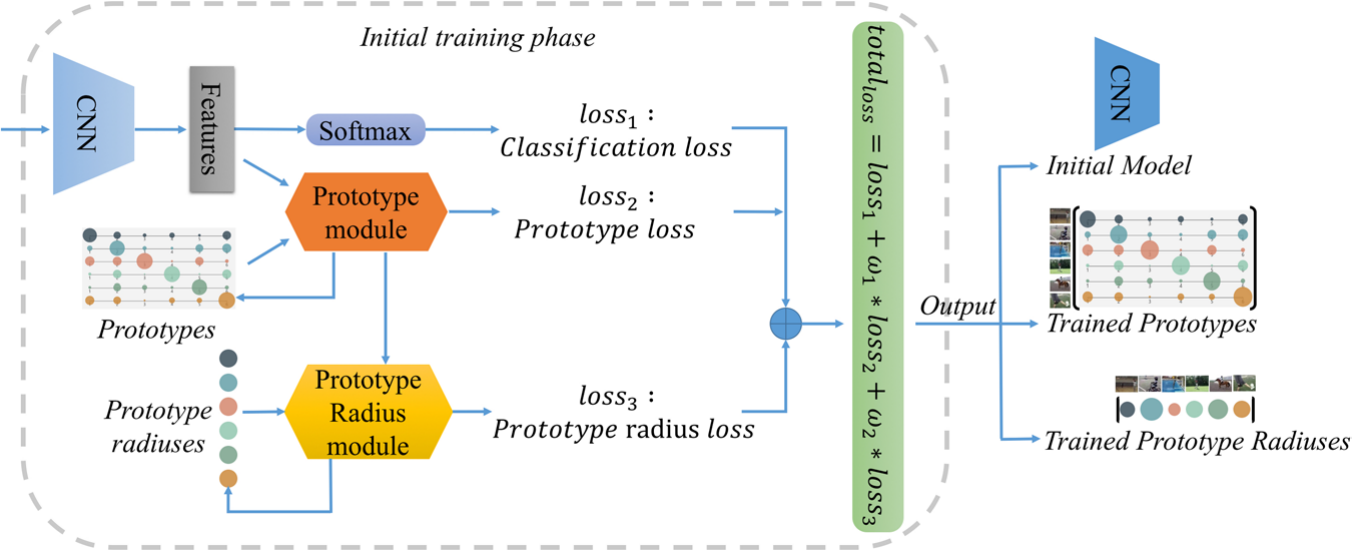
Structure of the prototype-based open deep network (P-ODN) in the initial training phase. The CNN takes knowns as input, and a classification loss is applied to train the initial classification model. Two major modules, the prototype module and the prototype radius module. The prototype module takes the features extracted by CNN as input and learns prototypes for categories. And the prototype radius module takes the prototype-based distances as input, where the distances are calculated in the prototype module, and learns the scope of each category prototype. Finally, the initial training phase outputs an initial model, trained prototypes and prototype radiuses for the incremental training phase later.

The structure of prototype-based open deep network (P-ODN) in the initial training phase is shown in Fig. 3. Basically, this phase includes two major modules: first, a *prototype module* is applied to learn prototypes of categories based on the prototype learning. Second, to guarantee each prototype of the category in a certain range, a *prototype radius module* is proposed. Each category will learn a prototype radius to further restrict the scope of features derived by the model. Three kinds of losses are applied to train prototypes and prototype radiuses. First, we apply the cross-entropy loss to train the classification capacity of the neural networks, which we denote as $los{s}_{1}$:

1 $$los{s}_{1}=-\frac{1}{S}\mathop{\sum }\limits_{i=1}^{S}\,[labe{l}_{i}\ast log(softmax(f))]$$

where $S$ is the batch size, ${f}_{i}$ is the feature of the $i$th sample in the batch, and $labe{l}_{i}$ is the ground truth. Second, the prototype loss, which is firstly proposed by^16^, is modified to apply in our framework to train the prototypes of knowns. Third, we propose the prototype radius loss, which guides the model to learn the radius scope of each known category.

The initial training phase outputs the initial model, trained prototypes and prototype radiuses, and they will be used later in the incremental training phase.

Major modules (*Detecting Unknowns* and *Updating Network*) of P-ODN in the Incremental training phase are shown in Fig. 2. The initial model trained in the Initial training phase extracts the features of the incremental training set here. We will simply review a triplet thresholding method of unknowns detection. And the method is modified to be applicable in the P-ODN to detect the unknowns based on the distance metric of the features and the trained prototypes. Then, a new distances based weights initialization method is introduced to initialize the weights of new category predictors in the *Updating Network module*. After the initialization of new weights, few manual labeled samples are used to fine-tune the model. New categories are incorporated in the current model continuously. At the end of this phase, a final model that can handle both knowns and unknowns is trained.

### Prototype module

**Figure 4:**
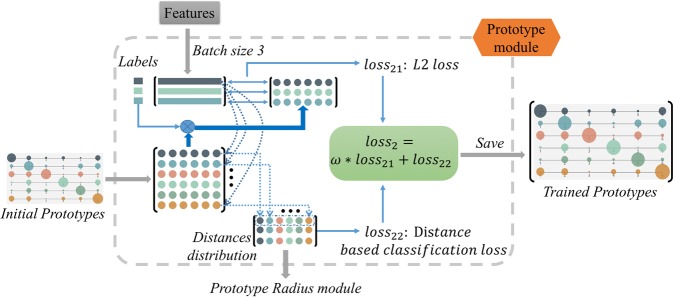
The illustration of Prototype Module. Different colors represent different categories in the figure. To give an explicit explanation of the process, we assume the batch size is 3 as shown in the figure. The corresponding prototypes of categories are chosen according to the labels of features. Then an L2 loss is applied to indicate the prototypes to learn. Simultaneously, a distance distribution matrix is calculated to train the capacity of the prototypes with the distance-based classification loss.

Figure 4 illustrates the algorithm of training the prototypes to represent the centers of knowns. Since prototype learning has shown its effectiveness in increasing the inter-class variation^16^, we introduce the prototype learning into open set recognition tasks and further use prototypes to detect unknowns.

Specifically, a $N\times N$ prototype matrix is initialized with zeros, where N is the category number of knowns. Each row of the prototype matrix, shown in different colors in Fig. 4, represents the prototype (or center) of each known category. The prototype loss ($los{s}_{2}$) is applied to acquire the trained prototypes which vary greatly in different categories. The prototype loss consist of a L2 loss ($los{s}_{21}$) and a distance-based classification loss ($los{s}_{22}$). Then the two losses are combined with a weight argument $\omega$:

2 $$los{s}_{2}=\omega \,\ast \,los{s}_{21}+los{s}_{22}$$

#### The L2 loss

*S* prototypes are chosen according to the label of features, where $S$ is the batch size of the CNN networks. To give an explicit explanation of the process, we assume the batch size is 3 as shown in Fig. 4. The L2 loss is applied to the chosen prototypes and the features to guide prototypes to learn the characters of the features:

3 $$los{s}_{21}=-\,\frac{1}{2S}\,\mathop{\sum }\limits_{i=1}^{S}\,{({f}_{i}-{p}_{i})}^{2}$$

where ${f}_{i}$ is the feature of the $i$th data sample in the batch and ${p}_{i}$ is the corresponding prototype.

#### The distance-based classification loss

As the prototypes and features are trained jointly, simply applying the L2 loss to make prototypes similar to the features would be unstable. The prototypes would be easily misled by some outliers of the training data samples. We add the distance-based classification loss to improve the classification capacity of the prototypes and increasing the penalty of misclassification samples, which helps to learn more stable and characteristic prototypes of categories.

As shown in Fig. 4, the Euclidean distance of each feature and each category prototype is calculated to get a distance distribution matrix $D$:

4 $${D}_{ij}=\frac{1}{\parallel {f}_{i}-{p}_{j}{\parallel }_{2}^{2}+\varepsilon }$$

where $i=\{1,\cdots ,S\}$ and $j=\{1,\cdots ,N\}$. We take the reciprocal of distances between features and prototypes here to make features near to the prototypes get larger probability value. And $\varepsilon =0.001$ is applied to avoid dividing by zero. So classification can be implemented by assigning labels according to the largest value in each row of $D$. Then the cross-entropy loss is applied on $D$, the $los{s}_{22}$:

5 $$los{s}_{22}=-\,\frac{1}{S}\,\mathop{\sum }\limits_{i=1}^{S}\,[labe{l}_{i}\,\ast \,log(softmax(D[i,:\,]))]$$

where the $S$ is the batch size (the same as the row number of $D$), $labe{l}_{i}$ is the ground truth, and $D[i,:\,]$ denotes the $i$th row of $D$.

In this module, P-ODN learns the category prototypes by applying the $los{s}_{2}$, then the trained prototypes are saved which will be used in the Incremental training phase.

### Prototype radius module

The *prototype radius module* is a key part of the initial training phase which aims to restrict the values of prototypes to a certain range and learn the prototype radius of categories. The prototypes and the prototype radiuses are trained jointly by adding an L2 loss ($los{s}_{3}$) which can be regarded as a regularization item of the prototype learning.

**Figure 5:**
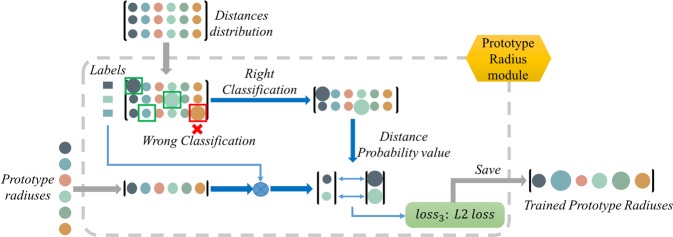
The illustration of Prototype Radius Module. To restrict the features to a certain range, the prototype radius module is applied. First, the correctly classified rows of the distance distribution matrix are chosen. Then the distance values of the corresponding categories are used to train the prototype radiuses with a L2 loss. This module can be regarded as a regularization item of the prototypes.

As shown in Fig. 5, a vector of each category prototype radius is initialized with zeros. The distance distribution matrix ($D$) calculated in the *prototype module* is inputted to the *prototype radius module*. Then the correctly classified probability scores of $D$ are chosen to guide the prototype radiuses to learn, the $los{s}_{3}$:

6 $$los{s}_{3}=-\,\frac{1}{2T}\,\mathop{\sum }\limits_{t=1}^{T}\,{({r}_{t}-{d}_{t})}^{2}$$

where $t$ is the number of correctly classified distance probability values, ${d}_{t}$ is the $t$th correctly classified distance probability value (the largest score of distance distribution row), and ${r}_{t}$ is the category prototype radius which is chosen according to the labels of samples.

The prototype radiuses act like a memory unit of the distance probability values of the correctly classified samples. It is updated according to the correctly classified samples continuously. Simultaneously it stores the distance probability value information of the previous correctly classified samples. So the *prototype radius module* integrates the long-term information to restrict the distance distribution value to a certain range and indirectly restricts the scope of the features extracted by the model. The use of long-term information improves a lot especially in the temporal stream of action recognition.

In this module, P-ODN learns the category prototype radiuses by applying the $los{s}_{3}$ and the trained prototype radiuses are also saved. Note that the total loss of the initial training phase goes as:

7 $$total\_loss=los{s}_{1}+{w}_{1}\,\ast \,los{s}_{2}+{w}_{2}\,\ast \,los{s}_{3}$$

where ${w}_{1}$ and ${w}_{2}$ are weight arguments, we set as $0.1$ and $0.01$ in our experiments.

The prototype radiuses are trained jointly with the prototypes, which play an important role as a regularization item to train the prototypes.

### Detecting unknowns

In our previous work^17^, we proposed a multi-class triplet thresholding method to detect the unknowns. Basically, a triplet threshold ($[\eta ,\mu ,\delta ]$) per category is calculated, i.e. accept threshold $\eta$, reject threshold $\mu$ and distance-reject threshold $\delta$. The triplet threshold $[{\eta }_{i},{\mu }_{i},{\delta }_{i}]$ of category $i$ is calculated as

8 $${\eta }_{i}=\frac{1}{{X}_{i}}\,\mathop{\sum }\limits_{j=1}^{{X}_{i}}\,{F}_{i,j}$$

9 $${\mu }_{i}=\varepsilon \ast {\eta }_{i}$$

10 $${\delta }_{i}=\rho \ast \frac{1}{{X}_{i}}\,\mathop{\sum }\limits_{j=1}^{{X}_{i}}\,({F}_{i,j}-{S}_{i,j})$$

where the ${F}_{i,j}$ and ${S}_{i,j}$ are the maximal and the second maximal confidence values of the $j$th correctly classified sample of category $i$. ${X}_{i}$ is the number of the correctly classified sample set ${{\mathscr{X}}}_{i}$ of category $i$. $\varepsilon$ and $\rho$ are empirical parameters.

**Figure 6:**
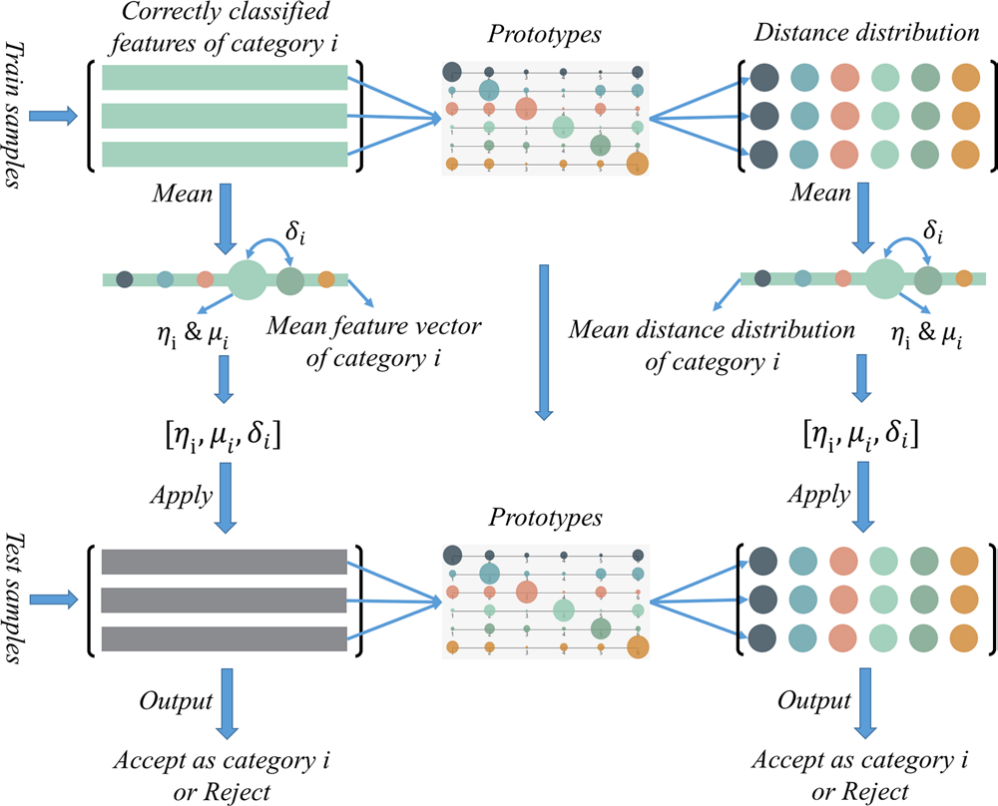
The illustration of Detecting Unknowns in the P-ODN. The first column can be viewed as our previous version in^17^. In the P-ODN, the distance distribution matrix is calculated by using the prototypes. Thresholds calculated to the mean distance distribution are then applied on the distance distribution of the test samples to detect unknowns.

A data sample is classified as category label *l* only if the index of its top confidence value is *l* and the value is greater than ${\eta }_{l}$. And a sample is regarded as unknowns when all of its confidence value is below $\mu$. The threshold $\delta$ is applied to help detect unknowns in hard samples, which lie between $\eta$ and $\mu$. The statistical properties of $\delta$ include correlation information between the two categories, which is a simple way of using the inter-class relation information in the activation level. If the distance is large enough, then we accept the data sample as category label *l*. The process of unknowns detection is shown in the first column of Fig. 6.

Unlike the previous version of unknowns detection in^17^, we modify the method to be applicable in the P-ODN framework to apply a more robust unknowns detection algorithm based on the distance metric. Figure 6 shows the comparison of detecting unknowns between our previous work ODN^17^ and the P-ODN. Instead of calculating the triplet thresholds based on the mean feature vectors, the distance distribution matrix of features and prototypes is calculated first. Then the triplet thresholds are calculated for each category in the same routine while based on the mean distance distribution. While the triplet thresholds are acquired, the later unknowns detection processes are similar.

The insight of this improvement is that thresholds are calculated based on statistic results of the train samples in the previous version. The statistic results, mean feature vectors, are more easily affected by the outliers. While in the P-ODN, the features are transformed into a different feature space by calculating the distance distribution matrix. Then thresholds are calculated based on the mean distance distribution. We assume that in this way the thresholds are acquired by making use of the model information, instead of only getting from the statistic information of data sets, which is more robust. Because the distance distribution can be regarded as a projection of features under the guidance of prototypes, which are trained with the model.

### Updating network

After detecting the unknowns, manual labeling the unknown samples. Then these samples could be used to fine-tune the model. It has been discussed that retraining the entire system with the known data and new samples is time-consuming, computational resource wasting. And it is also easy to be over-fitting, because new categories are far short of training samples.

In our previous work^17^, an updating method by transferring knowledge from the trained model was proposed which helps to speed up the training stage and needs very few manually annotations. A brief retrospective of the method is given below.

In each iteration of the incremental training phase, a new category is incorporated into the current model, which is carried out by increasing the corresponding weight column in the classification layer of the networks. By initialization of the weight column as Formula (11), the knowledge of the previous model is kind of transferred to the new model.

11 $${w}_{N+1}=\alpha \frac{1}{N}\,\mathop{\sum }\limits_{n=1}^{N}\,{w}_{n}+\beta \frac{1}{M}\,\mathop{\sum }\limits_{m=1}^{M}\,{w}_{m}$$

In Formula (11), the current category number is $N$ and ${w}_{n}$ is the weight column of the *n*th category in the classification layer of the networks. And ${w}_{m}$ is the weight column of $M$ most similar categories measuring by the scores of features. $\alpha$ and $\beta$ are empirical parameters.

**Figure 7:**
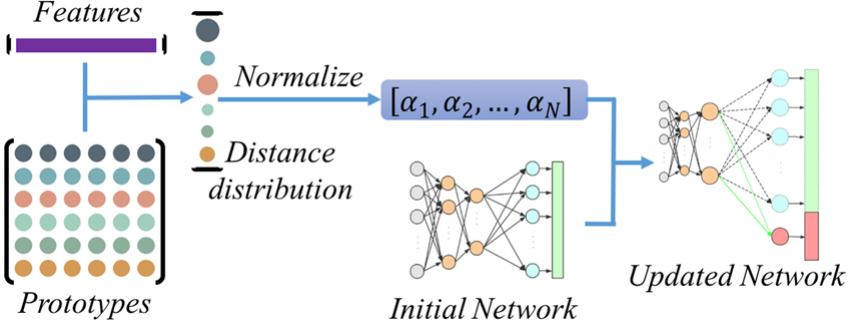
The illustration of distances based weights initialization. Distance distribution is calculated between prototypes and the new sample features. Then a weight distribution can be acquired by applying the mean normalization. Finally, weights of new predictors are initialized depending on weights of the initial networks according to the weight distribution.

In the P-ODN, a new weight column is also increased in the classification layer to incorporate the new category. Unlike the previous version, the distance distribution of the new category sample is calculated first. Then by applying the mean normalization, we can get the distribution $[{\alpha }_{1},{\alpha }_{2},\cdots ,{\alpha }_{N}]$, where $1={\sum }_{n=1}^{N}\,{\alpha }_{n}$, as shown in Fig. 7. The new weight ${w}_{N+1}$ is initialized as:

12 $${w}_{N+1}=\frac{1}{N}\,\mathop{\sum }\limits_{n=1}^{N}\,{\alpha }_{n}\ast {w}_{n}$$

where ${w}_{n}$ is the weight column of the *n*th category, and $N$ is the current category number.

The insight of this improvement is that more robust relations of the new category and the knowns are taken into account by applying the distances based weights initialization method. By initializing the new weights like Formula (12), the global knowledge, as well as the relation knowledge, can both be incorporated into the new model. First, each weight column is integrated to initialize the new weights, which guarantees new weights in the same distribution with knowns. And second, the distances based relation metric is much more robust than that in the previous work^17^ which is measured by comparing the scores in the features.

After the networks are updated, a few samples detected in the *Detecting Unknowns* module are used to fine-tune the model. As the new weights incorporate the knowledge of the previous model, the fine-tuning phase is much less complex and very soon. We also adopt the Allometry Training method and the Balance Training method, which are proposed in^17^, while fine-tuning the model. Specifically, different learning rates are embedded into the classification layer to force the new weights to learn at a faster rate. And we use the same few samples of each known and new category to avoid the greatest influence on the accuracy of the knowns. At the end of the Incremental training phase, the final model can classify both knowns and unknowns.

## Experiments

This section will first introduce the details of datasets and the evaluation schemes. Then, we describe the experiments setting and the exploration experiment.

### Datesets

To verify the effectiveness of P-ODN, we conducted experiments on four public datasets, including UCF11^18^, UCF50^19^, UCF101^20^ and HMDB51^21^. Note that we divide the datasets into knowns and unknowns to simulate the open world scenarios.

The UCF11 dataset contains $11$ action categories. For each category, the videos are grouped into $25$ groups with more than $4$ action clips in it. The video clips in the same group share some common features, such as the same actor, similar background, similar viewpoint, and so on.

The UCF50 dataset is an action recognition dataset with $50$ action categories, consisting of realistic videos taken from Youtube. For all the $50$ categories, the videos are grouped into $25$ groups, where each group consists of more than $4$ action clips.

The UCF101 dataset is one of the most popular action recognition benchmarks. It contains 13,320 video clips from $101$ action categories and there are at least $100$ video clips for each category.

The HMDB51 dataset is a large collection of realistic videos from various sources, including movies and web videos. It contains $6849$ clips divided into $51$ action categories, each containing a minimum of $100$ clips.

Compared with the very large dataset used for image classification, the dataset for action recognition is relatively small. Therefore we pre-trained our model on the ImageNet dataset^22^.

### Experiments setting

To simulate the open-world scenarios, we choose nearly half categories of each data set as knowns and the other half as unknowns, i.e. $6$ categories of UCF11 as knowns while the other $5$ as unknowns, $25$ categories of UCF50 as knowns while the other $25$ as unknowns, $50$ categories of UCF101 as knowns while the other $51$ as unknowns, and $25$ categories of HMDB51 as knowns while the other $26$ as unknowns. Then the training set of each dataset is divided into two subsets according to knowns and unknowns. The subset which contains knowns is the initial training set. A small subset is chosen from both the knowns and unknowns, which we guarantee that each category has at least $10$ samples, to form the incremental training set. Note that we use much less training samples and removing half labels of the training set to simulate the open world scenarios.

After initializing from the pre-trained ImageNet model for spatial and temporal streams, we conduct the initial training phase to train prototypes, prototype radiuses of categories, and the initial model jointly. Note that both the spatial stream and the temporal stream train their prototypes and prototype radiuses respectively. After prototypes and prototype radiuses for the two streams are trained, then triplet thresholds of both spatial and temporal streams are calculated on the initial training set based on the prototypes.

During the incremental training phase, we keep the same experiment setting as our previous work^17^ to give a convincing comparison. Basically, we update the networks when the number of any labeled new category goes to $5$, then this category is incorporated into the current model.

The experiments show that we use $53$ iterations (on average) to increase $51$ new categories while using dataset UCF101 ($27$ iterations for UCF50 to increase $25$, $7$ iterations for UCF11 to increase $5$ and $26$ for HMDB51 to increase $26$ new categories). So, on average, UCF101 needs to label $5.2$ ($53\times 5\div51=5.2$) samples ($5.4$ samples for UCF50, $7$ samples for UCF11 and $5$ samples for HMDB51) for each unknown categories. A more explict comparison is shown in the Table 1 between our previous ODN^17^ and P-ODN, it is obvious that we use the same number of labeled unknown samples in the P-ODN or even less.

**Table 1:** Sample number of manually annotated unknowns needed to increase a category on average.

Sample number	UCF11	UCF50	UCF101	HMDB51
ODN	7	5.8	5.39	5.57
P-ODN	7	5.4	5.2	5

However, for closed set recognition, using UCF101 as an example, the training list of UCF101 split1 has $9537$ data samples of $101$ categories. On average, each category, half of the categories corresponding to the known categories and half to the unknown categories, needs $94.4$ ($9537\div101=94.4$) annotated samples. We use much less samples of unknowns in the open set setting.

We also conduct the closed set recognition experiments as our baseline using the same sample size as the open set setting. The results of experiments in a closed set setting are much less than those of our P-ODN while both using insufficient unknown samples. So, P-ODN needs much fewer human annotations then the closed set recognition, and can achieve better performance. Worth to mention that, P-ODN suits the real-world scenarios, while the closed set recognition can not handle these tasks.

Finally, we give a detailed introduction to the action recognition backbone we use in our experiments. We apply the TSN^2^ model with images and optical flows as inputs. For the extraction of optical flow, we choose the TVL1 optical flow algorithm^23^ implemented in OpenCV with CUDA. We resize all input images and optical flows to $340\times 256$, and then use the fixed-crop strategy^24^ to crop a $299\times 299$ region from images or their horizontal flips. And we report the results on the backbone of the Inception-Resnet-v2 network^25^. We initialize network weights with pre-trained models from ImageNet^22^. The network weights are trained using the mini-batch stochastic gradient descent with momentum (set to $0.9$) and the batch size is set to 32. The learning rate is initialized as 0.01 and decreases to its $\frac{1}{10}$ at the 20, 000th, 30,000th and 40,000th iteration respectively. The whole training procedure stops at 50, 000 iterations. We use the Tensorflow toolbox^26^ to implement our algorithm.

### Exploration experiments

#### Benefits from prototypes

To illustrate the improvement of prototypes, we firstly conduct an exploration experiments on UCF101 with GoogLeNet^27^.

**Figure 8:**
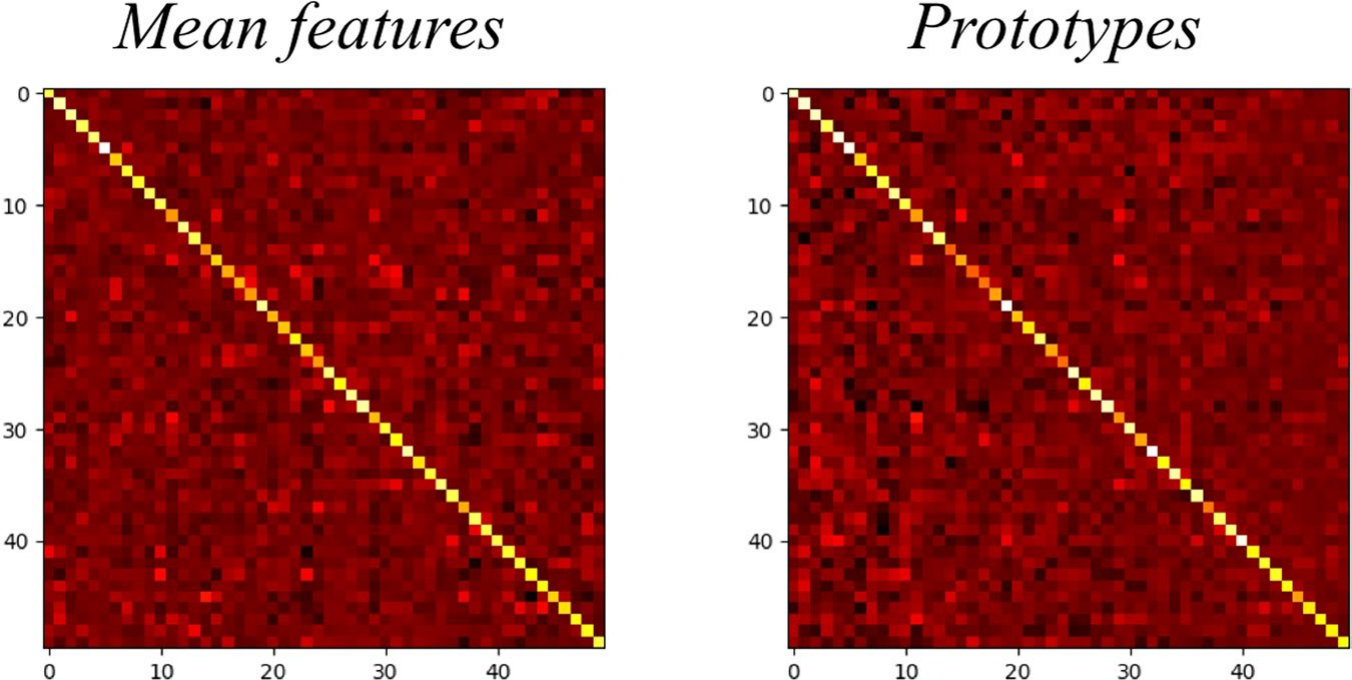
Heat map of mean features and prototypes of knowns. Prototypes have a much stronger response on the correctly classified values, for the diagonal line is much brighter while the upper and lower triangular matrices are much darker.

We visualize the heat map of the mean features and prototypes of the knowns, as shown in Fig. 8. We can see that prototypes have a much stronger response on the correctly classified values, for the diagonal line is much brighter while the upper and lower triangular matrices are much darker.

**Figure 9:**
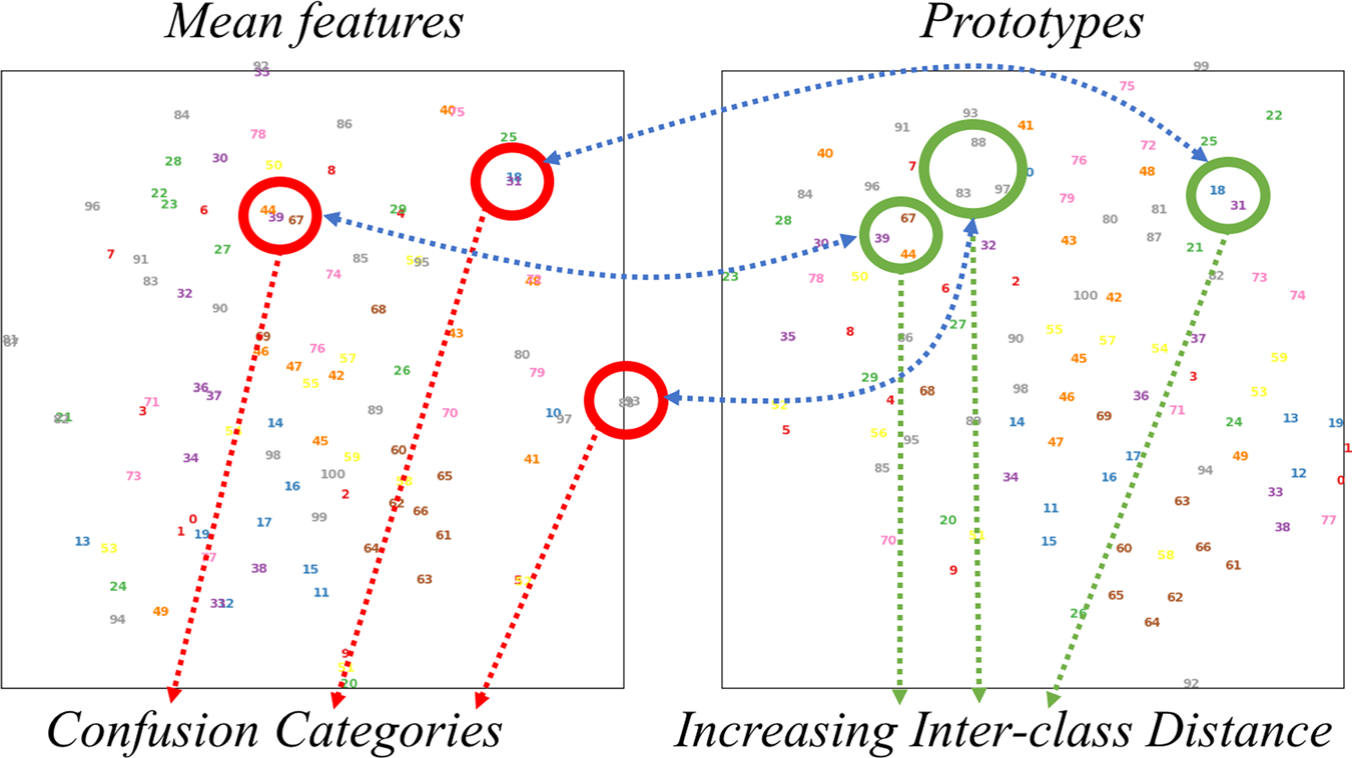
T-SNE visualization of mean features and prototypes. Each colored number represents a mean feature or a prototype of a certain category. The left visualization of the mean features has more confusion categories which the inter-class distances are short, while the prototypes can better handle the confusion categories as shown in the right sub-figure.

As shown in Fig. 9, we reduce dimensions of the mean features and the prototypes, and visualize them by applying t-SNE. In the figure, each colored number represents one mean feature of a certain category or one prototype of a certain category. The left visualization of the mean features has more confusion categories which the inter-class distances are short, while the prototypes can better handle the confusion categories as shown in the right sub-figure.

The comparison shows that the prototypes are more suitable for representing category centers. Because the prototypes have a stronger response on the collected classified values and longer inter-class distances. The two advantages help to complement more robust unknowns detection and guide much better features training.

## Results and Analysis

In this section, we report the experimental results and give the analysis of results.

### Evaluation of detecting unknowns

In this subsection, we aim to evaluate the unknowns detection performance of our P-ODN on UCF11, UCF50, UCF101 and HMDB51. As mentioned before, we conduct this evaluation at the end of the initial training phase as *Evaluation phase 1*. The experimental results are summarized in Table 2. The first row of the results is the performance of OSDN proposed in^14^, we conduct the method on the action recognition task. The second row is the performance of our previous work^17^. And the third row is the performance of P-ODN with *prototype module* only, the last row is P-ODN with both *prototype module* and *prototype radius module*. We can see P-ODN with both *prototype module* and the *prototype radius module* improves the ODN by $\mathrm{2.11 \% }$ on UCF11, $\mathrm{7.24 \% }$ on UCF50, $\mathrm{2.85 \% }$ on UCF101 and $\mathrm{3.66 \% }$ on HMDB51.

**Table 2:** Unknowns detection results of P-ODN.

F-score	UCF11	UCF50	UCF101	HMDB51
OSDN^14^	82.59%	75.34%	72.1%	50.31%
ODN^17^	87.39%	74.91%	73.35%	63.70%
P-ODN	89.12%	80.14%	75.45%	66.79%
P-ODN + radius	**89.50%**	**82.15%**	**76.2%**	**67.36%**

Unknown detection based on the prototypes are more robust. First the prototypes are more discriminable than mean features. Second, the prototypes can guide the features to be trained better, which helps to improve the intra-class compactness and inter-class distance of the feature representation. We also learn the triplet thresholds based on the prototypes which would contain the knowledge of the model itself. Then the much discriminable features and the model-based triplet thresholds both lead to a great improvement in the performance of unknowns detection.

### Evaluation of classification on both knowns and unknowns

In this subsection, we aim to evaluate the classification performance of our P-ODN on UCF11, UCF50, UCF101 and HMDB51. We conduct this evaluation at the end of the incremental training phase as *Evaluation phase 2*, the final classification accuracy of both knowns and unknowns is viewed as the most important performance indicator of open set recognition tasks. The experimental results are summarized in Table 3. First, we carry out the closed set recognition experiments while using the same quantity of samples as those of our open set setting. Under the closed set setting, all training samples should have labels, so we provide labels of both knowns and unknowns. The result is shown in the first row as our baseline. The rest of Table 3 are results under the open set setting. The second row is the result in our previous work^17^, and we add the experiment on UCF11 here, since we did not use UCF11 in the previous work. The last row is P-ODN with both *prototype module* and *prototype radius module*, which achieves the best performance. We can see P-ODN finally improves the ODN by $\mathrm{0.3 \% }$ on UCF11, $\mathrm{2.42 \% }$ on UCF50, $\mathrm{2.57 \% }$ on UCF101 and $\mathrm{3.08 \% }$ on HMDB51. And furthermore, P-ODN finally improves the baseline by $\mathrm{10.21 \% }$ on UCF11, $\mathrm{11.2 \% }$ on UCF50, $\mathrm{6.63 \% }$ on UCF101 and $\mathrm{4.51 \% }$ on HMDB51. A more explicit illustration can be seen in Fig. 11. Each sub-figure is corresponding to a data set, UCF11, UCF50, UCF101 and HMDB51. Take the sub-figure 11(a) as an example, we compare the accuracy of both knowns and unknowns on UCF11 with four methods, which are baseline, ODN^17^, P-ODN and P-ODN with radius (P-ODN with both *prototype module* and the *prototype radius module*). The light gray bar denotes the accuracy of knowns of baseline, while the dull gray denotes the accuracy of unknowns of baseline. The light blue line denotes the accuracy of knowns of ODN, while the dark blue denotes the accuracy of unknowns of ODN. And so on, the light green denotes the knowns of P-ODN and dark green denotes the unknowns of P-ODN. The light red and the dark red denote the knowns and unknowns of P-ODN with radius respectively.

**Table 3:** Recognition results of P-ODN.

TOP1 Acc.	UCF11	UCF50	UCF101	HMDB51
baseline	85.1%	84.95%	72.01%	44.58%
ODN^17^	94.91%	93.73%	76.07%	46.01%
P-ODN	94.9%	95.16%	77.21%	47.84%
P-ODN + radius	**95.31%**	**96.15%**	**78.64%**	**49.09%**

**Figure 10:**
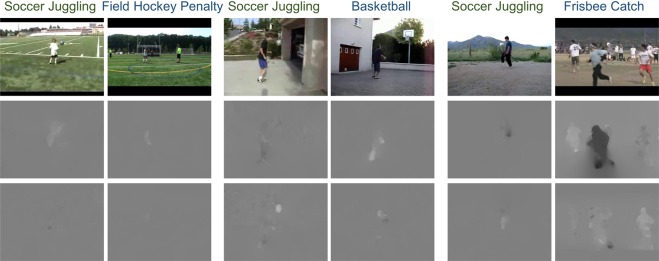
The new category *SoccerJuggling* is mainly initialized with three known categories *FieldHockeyPenalty*, *Backetball* and *FrisbeeCatch* according to the distances between them. As visualized in the figure, *SoccerJuggling* might share the similar scenes with *FieldHockeyPenalty*, and the motions of human while playing basketball, frisbee catching and soccer juggling share many same motion trajectories and motion units like running and jumping. The new category learns from the knowns by initializing the weights with the trained weights of the knowns, which enables the new categories to be trained with few samples.

**Figure 11:**
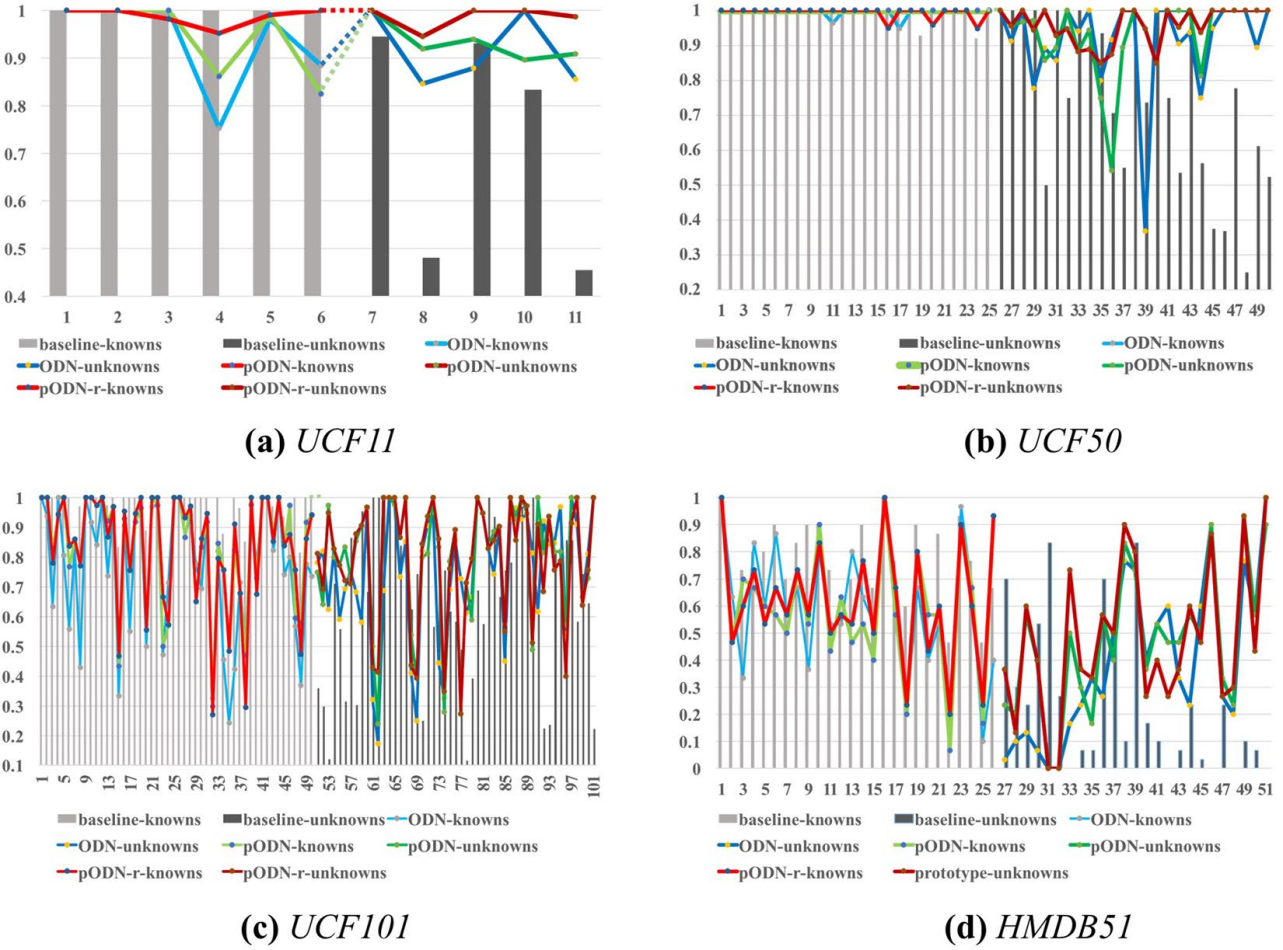
Comparison of baseline method, ODN^17^, P-ODN and P-ODN with radius on category accuracy of UCF11, UCF50, UCF101 and HMDB51 in real-world scenarios. Each sub-figure (**a**) is corresponding to a data set, taking the sub-figure as an example. Best viewed in color. The light gray bar denotes the accuracy of knowns of baseline, while the dull gray denotes the accuracy of unknowns of baseline. The light blue line denotes the accuracy of knowns of ODN, while the dark blue denotes the accuracy of unknowns of ODN. And so on, the light green denotes the knowns of P-ODN and dark green denotes the unknowns of P-ODN. The light red and the dark red denote the knowns and unknowns of P-ODN with radius respectively. The sub-figure (**b**) shows the results of UCF50, the sub-figure (**c**) shows the results of UCF101 and the sub-figure (**d**) shows the results of HMDB51.

We can see that while using the baseline method, the knowns which are trained with abundant data samples can achieve a much better performance than the unknowns which are trained with insufficient data samples with labels. Our methods can improve greatly on unknowns while using insufficient samples. Though, the performance on the knowns may decrease slightly, since the fine-tuning phase incorporates new data continuously. The known category *BrushingTeeth* decays slightly when adding a similar category *ShavingBeard*. Simultaneously, benefiting from transferring knowledge of the similar categories which share the basic motion unit of actions, the new categories can be trained with few samples by using our method, as shown in Fig. 10. The compromise is worth further studying, and we would focus on the trade-off issue in our future work. Finally, in Fig. 11, we can see P-ODN with radius is generally above the other methods, which achieves the best performance. Note that, different from the baseline method which is provided with all labels beforehand, the other three methods should detect the unknowns first, then manual labeling the unknowns. Therefore, open set recognition is more realistic than the closed set setting.

## Conclusion

This paper proposed a prototype-based Open Deep Network (P-ODN) for open set recognition. We introduce prototype learning into open set recognition tasks by training prototypes of categories and prototype radiuses with a *prototype module* and a *prototype radius module*. Then a distance metric method is applied to detect unknowns, which is based on the prototypes and more robust. In the incremental training phase, a distances based weights initialization method is employed to fast acquire the knowledge of the model and speed up the fine-tuning process. Experimental results show that, our P-ODN can effectively detect and recognize new categories with little human intervention and achieve state-of-the-art performance on UCF11, UCF50, UCF101 and HMDB51 datasets.

In this paper, we have proved the importance of more discriminable centers (or prototypes) on the open set recognition tasks. More characteristic features that have a larger margin among categories will further improve the performance of unknowns detection. Besides, method^28^ utilizes GAN to generate unknown samples and uses them to train the neural networks also has the potential to improve the recognition performance of unknowns. In the future work, we will conduct more experiments as mentioned above to further improve the performance of open set recognition.

## Data Availability

The datasets generated during and/or analysed during the current study are available from the corresponding author on reasonable request.
